# The OBTAINS study: A nationwide cross-sectional survey on the implementation of extended or continuous infusion of β-lactams and vancomycin among neonatal sepsis patients in China

**DOI:** 10.3389/fphar.2022.1001924

**Published:** 2022-10-10

**Authors:** Pengxiang Zhou, Yinchu Cheng, Guangna Cao, Yan Xing, Suodi Zhai, Xiaomei Tong, Kehu Yang

**Affiliations:** ^1^ Evidence Based Medicine Center, School of Basic Medical Sciences, Lanzhou University, Lanzhou, China; ^2^ Key Laboratory of Evidence Based Medicine and Knowledge Translation of Gansu Province, Lanzhou, China; ^3^ Department of Pharmacy, Peking University Third Hospital, Beijing, China; ^4^ Institute for Drug Evaluation, Peking University Health Science Center, Beijing, China; ^5^ Department of Pediatrics, Peking University Third Hospital, Beijing, China

**Keywords:** extended infusion, continuous infusion, β-lactams, vancomycin, neonatal sepsis, cross-sectional survey

## Abstract

**Background:** Dosing strategies of β-lactams and vancomycin should be optimized according to pharmacokinetic/pharmacodynamic principles. However, there is no available data indicating the implementation of extended infusion (EI) or continuous infusion (CI) administration in the management of neonatal sepsis.

**Methods:** A nationwide cross-sectional survey was conducted and the pediatricians from 31 provinces in China were enrolled. A multidisciplinary team created the questionnaire, which had three sections and a total of 21 questions with open- and closed-ended responses. The survey was then conducted using an internet platform in an anonymous way. The data was eventually gathered, compiled, and examined. To identify the risk factors associated with the implementation of EI/CI, logistic regression was carried out.

**Results:** A total of 1501 respondents answered the questionnaires. The implementation of EI/CI of β-lactams and vancomycin were only available to one-third of the respondents, and the prolonged strategy was primarily supported by guidelines (71.25%) and advice from medical specialists (55.18%). A significant fraction (72.94%–94.71%) lacked a strong understanding of the infusions’ stability. Additionally, it was discovered that more frequent MDT discussions about antibiotic use and the appropriate time pediatricians worked in the neonatal ward were associated with an increase in the use of the EI/CI strategy.

**Conclusion:** The EI/CI strategy in neonatal sepsis was not well recognized in China, and it is necessary to establish a solid MDT team with regularly collaborates. In the near future, guidelines regarding prolonged infusion management in neonatal sepsis should be developed.

## Introduction

The management of neonatal and pediatric sepsis remains a significant and challenging public burdensome health issue on a worldwide scale (Ruth et al., 2014; Weiss et al., 2015; Shane et al., 2017). Globally pooled data revealed that the case fatality rate of pediatric sepsis was approximately 31.7% in developing countries and 19.3% in developed countries (Tan et al., 2019), and the mortality of neonatal sepsis ranged from 11% to 19% (Fleischmann-Struzek et al., 2018). Nevertheless, current guidelines and opinions recommended that dosing strategies of antibiotics should be optimized according to pharmacokinetic/pharmacodynamic (PK/PD) principles (Rhodes et al., 2017; Weiss et al., 2020) and encouraged to provide individualized antimicrobial dosing approaches for special populations whenever possible (Hartman et al., 2020; Tu et al., 2021; Cao et al., 2022) given the scarcity of antibiotic agents in the pediatric population and the rise in antibiotic resistance.

In neonatal sepsis, the choice of antibiotic is complex and should be based on clinical syndrome, underlying disease, drug intolerances, and local pathogen susceptibility (Plunkett and Tong, 2015). β-lactams and vancomycin are two of the most frequently utilized antimicrobials due to their broad range of activity and broad therapeutic index (Plunkett and Tong, 2015; Phe et al., 2020; Burgunder et al., 2022). β-lactams, the cornerstone of the empirical antibacterial therapy in neonatal sepsis owing to their rare toxicity with only a few exceptions (Muller et al., 2018), mainly include three categories: penicillins (e.g., amoxicillin, ampicillin, and oxacillin, et al.), cephalosporins (e.g., cefazolin, cefuroxime, and ceftriaxone et al.) and carbapenems (e.g., meropenem, imipenem-cistatin, and ertapenem, et al.). Vancomycin, one of the most frequently prescribed glycopeptides in neonatal infections, is recommended to cover vascular catheter-associated coagulase-negative staphylococci and Meticillin resistant *Staphylococcus aureus*(Jacqz-Aigrain et al., 2015; Minotti et al., 2021).

Generally, PK refers to the study of concentration changes of a drug over a given time period, and PD describes the relationship between PK exposure and pharmacological effect (Abdul-Aziz et al., 2015). As time-dependent antibiotics, extended infusion (EI) or continuous infusion (CI) administration of β-lactams could theoretically lead to improved PK/PD profiles when the concentration remains above the minimum inhibitory concentration (MIC) of the causative pathogen (fT > MIC) for a longer duration (Rizk et al., 2017; Veiga and Paiva, 2018). Vancomycin could also theoretically better meet PD targets when provided as prolonged administration (DiMondi and Rafferty, 2013; Gwee et al., 2014; Alonso-Moreno et al., 2021). Although EI/CI administration for critically ill patients has been suggested by clinical practice guidelines (Bretonnière et al., 2015; Egi et al., 2021; Evans et al., 2021), there lacks convincing evidence demonstrating its superiority compared with a short-term infusion or bolus dosing in children (Zhou et al., 2021), and pediatricians seem to be not knowledgeable of it or to apply it frequently in clinical practice. There are currently no data available demonstrating the practical use of EI/CI approaches in the management of neonatal sepsis in China.

This study is a subset of a national survey titled “Current status of drug optimization for the application of β-lactams antibiotics and vancomycin in the treatment of neonatal sepsis in China” (OBTAINS study). We aimed to conduct a cross-sectional survey of pediatricians in China to characterize the current implementation of EI/CI-lactams or vancomycin in neonatal sepsis and discover its potential factors.

## Methods

### Study design and participants

A nationwide cross-sectional survey was conducted to assess the clinical administration of EI/CI of β-lactams or vancomycin in neonatal sepsis in China using an online questionnaire. The target population was pediatricians who had experience in neonatal sepsis management, regardless of their pediatric secondary specialty in both private and public hospitals in China. A convenient sampling approach was applied to enroll participants throughout 31 Chinese provinces from April 22 to June 3, 2022. The participants received invitations to answer the questions through the link to the questionnaire platform *via* social media (WeChat group) or E-mail. Weekly reminders were sent as needed. Participation was anonymous and unpaid.

The study was performed in accordance with the Declaration of Helsinki and approved by the Peking University Third Hospital Medical Science Research Ethics Committee (M2022245).

### Questionnaire development

The questionnaire, which is a component of the OBTAINS study, consists of three sections, comprising a total of 21 questions with open- and closed-ended answers. The purpose of first section (7 questions) aimed to collect the demographic information of the respondents. Their clinical practice experience in neonatal wards was investigated in the second section (4 questions). Next, if the respondents had experience in EI/CI of β-lactams (penicillins: benzylpenicillin, amoxicillin, ampicillin, oxacillin, cloxacillin, amoxicillin-clavulanate, ampicillin-sulbactam, and piperacillin-tazobactam; cephalosporins: cefazolin, cefuroxime, ceftriaxone, cefotaxime, ceftizoxime, ceftazidime, ceftazidime-avibactam, cefoperazone-sulbactam, cefepime, and ceftaroline); latamoxef; and carbapenems: meropenem, imipenem-cistatin, biapenem, and ertapenem) and vancomycin in the management of neonatal sepsis, eight multiple-choice questions and two single-choice questions were required to be further answered. This section mainly reflected their experience with specific drugs with EI/CI administration, the method of obtaining this optimization method, the timing and reasons, the infusion time, the knowledge of antibiotic stability (amoxicillin, cloxacillin, ampicillin-sulbactam, piperacillin-tazobactam, cefotaxime, ceftazidime, cefepime, meropenem, imipenem-cistatin, and vancomycin), the loading dose, and the attitudes towards EI/CI administration based on the clinical observation. Surveys would skip to the end if respondents selected “no experience in EI/CI administration”.

Following a comprehensive literature review (Zhou et al., 2021), a multidisciplinary team of pediatricians, pharmacists, infectious disease specialists, and epidemiologists developed the questionnaire. The appearance validity of the questionnaire was then evaluated through an initial pilot test with eight specialists, including four pediatricians, two pharmacists, one epidemiologist, and one infectious diseases specialist. They were invited to provide feedbacks and suggestions on the understandability and logicality of the questions, the rationality of response time, and the validity of the survey platform before it was available on the Questionnaire Star platform (https://www.wjx.cn/). The English version of the questionnaire is available in Supplementary Table S1.

### Data collection and statistical analyses

The data were collected, cleaned and standardized by pairs of investigators (Z.P.X. and C.Y.C.) *via* Microsoft Excel 2019. Frequencies and proportions for categorical data were used primarily to summarize and describe participants’ demographic characteristics and their current clinical practice of EI/CI administration. A multivariable logistic regression model was applied to explore which demographic characteristics or clinical practice experience were associated with the implementation of EI/CI administration using the enter method for covariate selection. Odds ratios (ORs) with 95% confidence intervals (CIs) were calculated quantify the associations. All statistical tests were two-sided, and differences with *p* < 0.05 were considered statistically significant. The analyses were performed using IBM^®^ SPSS^®,^ Statistics 27.0, and Tableau (2022.1 version) was used to generate the figures.

## Results

### Respondent characteristics

**FIGURE 1:**
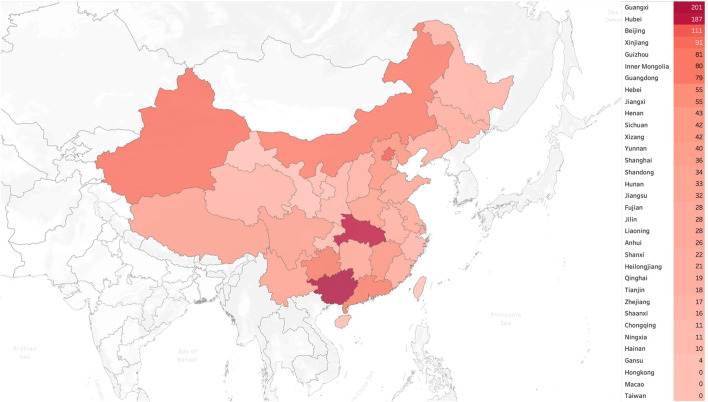
Geographical distribution of all respondents (*n* = 1501) in China.

**FIGURE 2:**
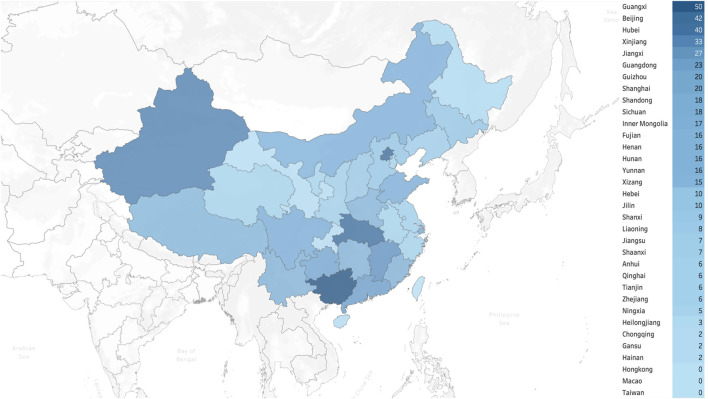
Geographical distribution of respondents with EI/CI experience (*n* = 473) in China.

A total of 1,501 pediatricians from 31 provincial administrative regions of China responded to the survey. The demographic characteristics of the participants are shown in Table 1, while Figure 1 and Figure 2 illustrate the geographic distribution of the responses.

**TABLE 1:** Respondents’ characteristics.

Questions	N	%
Age (years)		
<30	147	9.79
30–39	533	35.51
40–49	510	33.98
50–59	292	19.45
≥60	19	1.27
Sex		
Women	1136	75.68
Men	365	24.32
Technical title		
Junior	307	20.45
Intermediate	481	32.05
Associate-senior	400	26.65
Senior	313	20.85
Education		
Specialty	86	5.73
Bachelor	1016	67.69
Master	302	20.12
Doctor	97	6.46
Years of employment (years)		
<5	132	8.79
5–9	238	15.86
10–14	299	19.92
15–19	223	14.86
>20	609	40.57
Grade and level or hospitals		
Grade III general hospital	656	43.70
Grade III specialist hospital for women and children	317	21.12
Grade II general hospital	279	18.59
Grade II specialist hospital for women and children	109	7.26
Private general hospital	11	0.73
Private specialist hospital for women and children	5	0.33
Primary healthcare center (Grade I)	87	5.80
Other healthcare center	37	2.47
Department (ward)		
Neonatology	624	41.57
Neonatal intensive care unit	245	16.32
Pediatrics	375	24.98
Pediatric intensive care unit	8	0.53
Children’s healthcare unit	69	4.60
Other pediatric departments	180	11.99

### Clinical experience of neonatal sepsis management

The neonatal ward was where the most of the responders (54.56%, 819/1501) spent 10–12 months of the year at work. The most frequent ways of obtaining the experience of neonatal sepsis antibiotic management included pediatric team experience (69.22%, 1039/1501), classes from neonatal or pediatric specialists (79.21%, 1189/1501), and guidelines, consensus or medical textbooks (67.95%, 1020/1501). A total of 44.57% (669/1501) and 33.84% (508/1501) of the respondents gained experience *via* consulting clinical pharmacists and anti-infection specialists, respectively.

Unfortunately, 36.64% (550/1501) of the participants stated that their neonatal antibiotic management did not involve a multidisciplinary therapy (MDT) team. A total of 27.78% (417/1501) conducted MDTs by inviting formal medical consultations, whereas 16.59% (249/1501) addressed the antibiotic-related questions only through private communication with anti-infection specialists or pharmacists. Additionally, 31.78% (477/1501) of the pediatricians reported having no MDT discussion, whereas 41.71% (626/1501) said that MDT discussion was carried out depended on the necessity. Only 13.19% (198/1501) of the respondents had regular MDT discussions on varying frequencies.

### Extended infusion or continuous infusion of β-lactams and vancomycin implementation

Overall, only 31.51% (473/1501) of respondents reported that they had experience with EI/CI of β-lactams and vancomycin in neonatal sepsis. More than half indicated that they carried out EI/CI administration when the patients had a strong possibility of serious infections with multiple risk factors (55.81%, 264/473) or the blood cultures revealed that they were intermediate or resistant to β-lactams or vancomycin (53.07%, 251/473). Furthermore, 27.06% (128/1501) reported that they frequently used EI/CI therapy, and 40.17% (190/1501) claimed that the patients might benefit from EI/CI administration when initial antibiotics were ineffective.

The majority of respondents stated that they acquired the EI/CI approach *via* clinical practice guidelines (71.25%, 337/473) or lectures given by anti-infection specialists (55.18%, 261/473). Less than half of the participants indicated that they learned about the EI/CI approach *via* clinical pharmacists’ lectures (47.36%, 224/473), academic conferences (47.15%, 223/473) or their department guidance (45.45%, 215/473). Regarding infusion time, most of the respondents had experience with EI administration for 2–3 h (76.74% for β-lactams, 363/473; 81.61% for vancomycin, 386/473), whereas very few had experiences with CI administration (3.38% for β-lactams, 16/473; 8; 5.07% for vancomycin, 24/473) (Table 2).

**TABLE 2:** Experience in neonatal sepsis management.

Questions	n	%
Months in neonatal department (ward) per year		
<1	107	7.13
1–3	104	6.93
4–6	116	7.73
7–9	79	5.26
10–12	819	54.56
Seldom	276	18.39
The way accessing the experience of neonatal sepsis antibiotics management (N = 4636)		
Departmental team experience (e.g. internal SOP, clinical pathways or discussion)	1,039	69.22
Lectures or classes from neonatal or pediatric specialists	1,189	79.21
Published guidelines, consensus or medical textbook	1,020	67.95
Consultation or teaching by clinical pharmacists	669	44.57
Consultation or teaching by anti-infection specialists	508	33.84
Not accessible	211	14.06
MDT team in neonatal antibiotic use		
There is a well-established MDT team for neonatal sepsis in the hospital	144	9.59
MDT is performed through inviting formal medical consultations	417	27.78
Invite a familiar anti-infection specialist, clinical pharmacy or microbiologist through private communication	249	16.59
No MDT teams	550	36.64
Unclear	141	9.39
The frequency of MDT discussion in antibiotic use management		
Daily^#^	30	2.00
4–5 times weekly^#^	24	1.60
2–3 times weekly^#^	48	3.20
Weekly^#^	96	6.40
Depends on the clinical requirement	626	41.71
No MDT discussion	477	31.78
Unclear	200	13.32

* Note: multiple-choice questions; # regular discussion.

**FIGURE 3:**
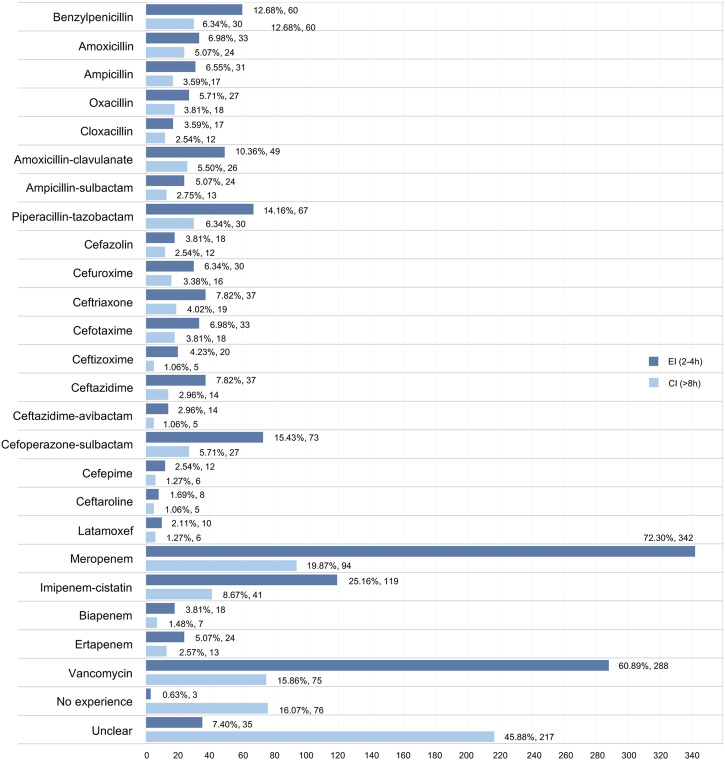
Participants’ responses about the experience of EI/CI approach in 24 antibiotics.

We investigated the experience of EI/CI administration in neonatal sepsis in 24 antibiotics, including 23 types of β-lactams and vancomycin. Overall, the EI (2–4 h) approach was conducted more frequently than the CI (>8 h) approach in all drugs of interest, while meropenem (25.16% in EI; 19.87% in CI) and vancomycin (60.89% in EI; 15.86% in EI) were the two drugs most likely to undergo EI/CI administration. Furthermore, piperacillin-tazobactam (14.16%) was the most commonly reported drug in the penicillin category with the EI/CI method, whereas cefoperazone-sulbactam (15.43%) was the most widely reported drug in the cephalosporin category (Figure 3).

In terms of loading dose of β-lactams or vancomycin, nearly half of the respondents (50.95%, 241/473) reported they would conduct a loading dose depending on the condition and severity of infection, whereas 17.12% (81/473) administered loading dose on a regular basis. The attitude towards EI/CI administration in neonatal physicians was investigated in this questionnaire, and approximately one-third (29.81%, 141/473) of the participants were convinced that they had achieved better clinical improvements. However, 32.35% (153/473) indicated they could not draw definite conclusions about the efficacy due to their limited experience, and 16.70% (79/473) reported there was inconsistency of efficacy based on previous cases. Only 4.65% (79/473) believed there was no clinical improvement in clinical efficacy following EI/CI administration (Table 3).

**TABLE 3:** EI/CI β-lactam and vancomycin implementation.

Questions	n	%
Do you have experience in EI/CI β-lactam and vancomycin		
Yes	473	31.51
No^#^	1,028	68.49
The reasons of EI/CI β-lactam and vancomycin (N = 473)*		
The patient has multiple risk factors and a strong probability of serious infections, such as multi-drug resistance infections	264	55.81
Blood culture results suggest that the bacteria are intermediate or resistance to β-lactam or vancomycin	251	53.07
Previous multiple antibiotics are ineffective, the efficacy may be improved by EI/CI administration	190	40.17
Routine EI/CI β-lactam and vancomycin experience	128	27.06
Unclear	28	5.92
The source of obtaining EI/CI administration (N = 473)*		
Clinical practice guidelines or expert consensus	337	71.25
Lectures from anti-infection specialists	261	55.18
Lectures from clinical pharmacists	224	47.36
Academic conferences	223	47.15
Clinical pathways or guidance within the department	215	45.45
Medical textbook or drug instructions	170	35.94
Experience from consulting other pediatricians	139	29.39
Unclear	15	3.17
Experience in the infusion time of EI/CI of β-lactams (N = 473)*		
Extended to 1 h	132	27.91
Extended to 2 h	259	54.76
Extended to 3 h	104	21.99
Extended to 4 h	69	14.59
Continuous to more than 8 h	16	3.38
Unclear	26	5.50
Experience in the infusion time of EI/CI vancomycin (N = 473)*		
Extended to 2 h	305	64.48
Extended to 3 h	81	17.12
Extended to 4 h	62	13.11
Continuous to more than 8 h	24	5.07
Unclear	81	17.12
Loading dose of β-lactam or vancomycin to achieve rapid plasma therapeutic concentrations in severe infected children (N = 473)		
Routine loading dose	81	17.12
Selective administration of loading dose according to the patient’s condition and severity of infection	241	50.95
No loading dose experience	124	26.22
Unclear	27	5.71
Attitude towards EI/CI administration according to clinical observation (N = 473)		
No definitive conclusions on its efficacy because of limited experience	153	32.35
It had achieved better clinical improvement	141	29.81
There was inconsistency in the efficacy based on various cases	79	16.70
No better clinical improvement was observed using EI/CI administration	22	4.65
Unclear	78	16.49

Note: # If the answer is no, the following questions are not required. * multiple-choice questions.

### Respondents’ knowledge about antibiotic stability

**FIGURE 4:**
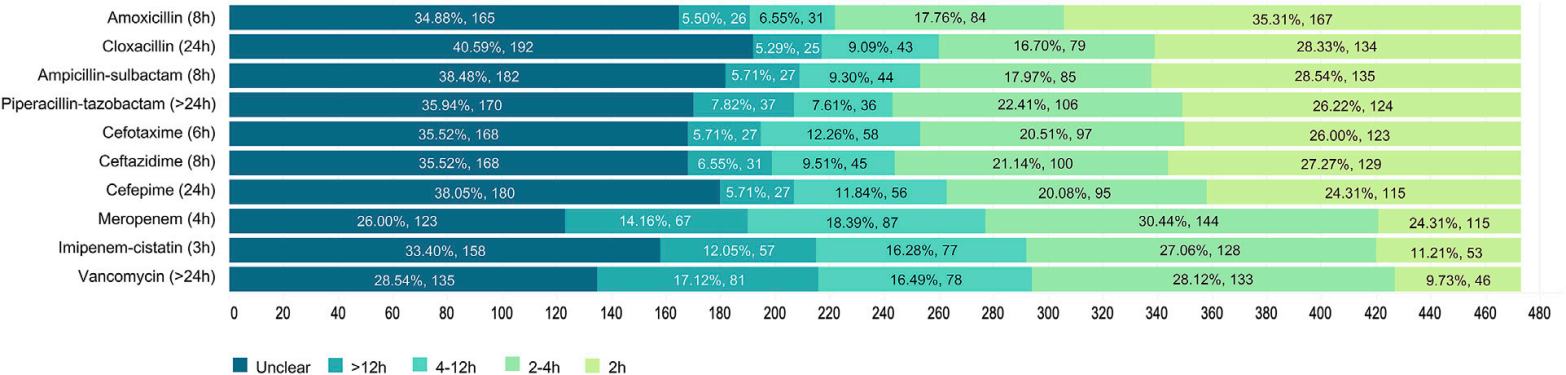
Participants’ responses about the duration of stability of antibiotics considered.

It was unknown how long intravenous β-lactams and vancomycin would remain stable. The most correctly recognized antibiotics in terms of infusion stability, according to published stability studies (Longuet et al., 2016; Loeuille et al., 2022), were imipenem-cistatin (27.06%), meropenem (18.39%), vancomycin (17.12%) and cefotaxime (12.26%), whereas less than 10% of the respondents could clearly identify the right stable time. Nearly 40% (34.88%–40.59%), 30% (26.00%–33.40%) and 28.54% of the participants stated that they were unclear about the stability of infusions in cephalosporins, carbapenems and vancomycin, respectively (Figure 4).

### Factors influencing extended infusion or continuous infusion implementation

The findings of the logistic regression indicated that the respondents with a master (*p* = 0.008) or Doctor degree (*p* = 0.011) and those who worked in a higher hospital grade (*p* = 0.004) or neonatal intensive care unit (*p* = 0.002) were more likely to administrate EI/CI. Additionally, individuals who spent 4–6 months (*p* = 0.003) in the neonatal department or worked in a well-established MDT team for neonatal sepsis (*p* = 0.023) with daily (*p* = 0.047), 2–3 times weekly (*p* = 0.044) or once weekly (*p* = 0.032) discussion were significantly linked to greater rates of EI/CI implementation (Supplementary Table S2).

## Discussion

To the best of our knowledge, this large nationwide survey represents the first cross-sectional study to investigate the implementation of EI/CI of β-lactams and vancomycin in neonatal sepsis in China. The findings could contribute to identify pediatricians’ current knowledge, clinical practice and attitude regarding this antimicrobial optimization strategy.

Generally, the findings of this survey were well represented by 1501 pediatric physicians in mainland China according to their demographic characteristics and clinical experience. It is worth mentioning that only approximately one-third of the respondents had access to EI/CI of β-lactams (most frequently meropenem, imipenem-cistatin, cefoperazone-sulbactam and piperacillin-tazobactam) and vancomycin for 2–3 h in most cases, with selective administration of a loading dose in critically infected neonates. Notably, the prolonged strategy was primarily supported by guidelines and consultations from medical specialists (such as anti-infection specialists, clinical pharmacists), but the attitude and clinical outcomes were of considerable uncertainty and wide variability among the respondents. A large proportion of respondents did not clearly know or provide accurate answers to the stability of nine kinds of β-lactams and vancomycin in our study. Due to unawareness of or uncertainty over the stability of infusion, pediatricians may consequently decide to abandon the EI/CI strategy.

Furthermore, the multivariate analysis findings demonstrated several potential factors, including the education level, the hospital grade, and the department or ward pediatricians worked in, that could be connected to the implementation of the EI/CI strategy in neonatal sepsis. Additionally, we discovered that an increase in the use of the EI/CI approach was linked to the appropriate time pediatricians worked in the neonatal department/ward, the well-developed MDT team for neonatal sepsis, and more frequent MDT discussion on antibiotic use. Several published studies reported that MDT intervention could benefit anti-infection management in the NICU (Hamza et al., 2022). Neonatologists are encouraged to develop and implement antibiotic utilization strategies in collaboration with surgeons (surgical prophylaxis, the management of surgical infections) (Robinson et al., 2020), infectious disease specialists (MIC and drug resistance), pharmacists (PK/PD theories, toxicities, interactions, and drug information), nurses (EI/CI care), and other NICU providers (Katz et al., 2021). Moreover, it is imperative to note that autonomous learning about antimicrobial stewardship in neonatal sepsis through lectures, guidelines and consultations was not substantially correlated with a higher proportion of EI/CI use in our analysis. Consequently, future quality improvement studies or antimicrobial stewardship practices should deliberately arise awareness, optimize processes, and utilize multidisciplinary resources for neonatologists (Li et al., 2022).

Previously, research on EI/CI strategies administered in intensive care units for adults demonstrated that even though optimized administration of β-lactams and vancomycin were universally acknowledged by intensive care specialists, limited fraction of them actually implemented these principles in clinical practice owing to the lack of knowledge and difficulties in accessing information (Dulhunty et al., 2011; Buyle et al., 2013; Tabah et al., 2015; Charmillon et al., 2016; Liebchen et al., 2020). However, only a few studies have focused on pediatric populations with severe infections and optimized antimicrobial dosing strategies (Knoderer et al., 2014). To our knowledge, few large sample-sized cross-sectional surveys have been carried out in China. It is of irreplaceability to further disseminate the knowledge about the PK/PD theory of prolonged strategy in time-dependent antibiotics, current best clinical evidence on efficacy and safety and implementation experience of EI/CI approaches (Tabah et al., 2015).

The studies indicated that the majority of neonatologists could recognize that antibacterial agents’ PK parameters (such as volume of distribution and drug clearance) are significantly altered in critically infected children (Hartman et al., 2020). However, the optimal antibiotic optimization in accordance with PK/PD theory remained multifaceted and poses particular challenges to neonatal pediatricians (Tängdén et al., 2017; Phe et al., 2020). The potential barriers to its implementation might be mainly attributed to the lack of pharmacological knowledge, the absence of an MDT team, and the lack of confidence in clinical practice owing to limited efficacy and safety evidence in pediatrics. Recent published systematic reviews have demonstrated that EI/CI had generally improved clinical outcomes and safety profiles compared with intermittent infusion strategy, with a greater probability of achieving the target (Costenaro et al., 2020; Alonso-Moreno et al., 2021; Zhou et al., 2021). It is undeniable that the EI/CI strategy has emerged as a promising option (De Waele et al., 2014), even though PK of antibiotics may be affected in critically ill neonates undergoing significant physiological alterations and the appropriateness of EI/CI administration remains further PK/PD or clinical evaluation data (Wang et al., 2020; Saito et al., 2021; Yonwises et al., 2021). A clinical practice guideline describing the methods and procedures for using EI/CI of β-lactams and vancomycin in neonatal sepsis management is consequently urgently required based on our study findings.

To further overcome these obstacles, specific implementation strategies should be developed accordingly (Correa et al., 2020). First, guidelines, research or clinical experience need to be disseminated to department administrators, pediatricians and patients’ families (Boland et al., 2019a; Boland et al., 2019b). Second, it is necessary to establish an MDT team regarding antibacterial agents, with at least anti-infection specialists, clinical pharmacists and microbiologists on board (Whalen et al., 2021). Third, the department’s policies, guidelines, or a trustable clinical decision support system may aid pediatricians in standardizing their EI/CI strategies and boosting their self-assurance (Euteneuer et al., 2019; Persad et al., 2021; Daphtary and Baloglu, 2022).

Nonetheless, there are several limitations in this study. Owing to resource constraints, we used a convenience sampling method to ensure that a specific number of results were available for individual province from China rather than conducting a strict stratified random sample across the nation. The questionnaire results might therefore be assumed representative. Additionally, the questions in the survey concerning clinical experience only reflect the impressions and attitudes towards the respondents other than the actual daily clinical practice. Finally, as a cross-sectional study, its causal association should be determined cautiously. The non-significant results in certain domains do not indicate a lack of association but may be due to limited sample size.

## Conclusion

In conclusion, this national survey demonstrated that the EI/CI strategy in neonatal sepsis was only recognized by one-third of Chinese neonatologists. To improve the implementation of antibacterial optimization, a solid MDT team should be developed with regularly collaborates. Guidelines regarding prolonged infusion management in neonatal sepsis would be beneficial for clinical practice and should be developed in the near future.

## Data Availability

The raw data supporting the conclusions of this article will be made available by the authors, without undue reservation.
