# Erratum: The OBTAINS study: A nationwide cross-sectional survey on the implementation of extended or continuous infusion of β-lactams and vancomycin among neonatal sepsis patients in China

**DOI:** 10.3389/fphar.2022.1120032

**Published:** 2022-12-16

**Authors:** Frontiers Production Office

**Affiliations:** Frontiers Media SA, Lausanne, Switzerland

**Keywords:** extended infusion, continuous infusion, β-lactams, vancomycin, neonatal sepsis, cross-sectional survey

Due to a production error, there was an error in the second Reviewer affiliation. Instead of “Yogan Khatri, Ann Arbor, United States”, it should be “Yogan Khatri, Cayman Chemical, United States”.

The publisher apologizes for this mistake. The original version of this article has been updated.

