# Supplementary material for: The OBTAINS study: A nationwide cross-sectional survey on the implementation of extended or continuous infusion of β-lactams and vancomycin among neonatal sepsis patients in China
Source: Front Pharmacol. 2022 Oct 10;13:1001924. doi: 10.3389/fphar.2022.1001924 (PMC9589050; doi:10.3389/fphar.2022.1001924)
Supplement: Supplementary file 1 [file DataSheet1.docx]

**Supplementary Materials**

**Supplementary material 1 Questionnaire about administration of β-lactams and vancomycin in neonatal sepsis in China**

| **Part 1 Basic Information** |
| --- |

1. How old are you? *(Single-choice question)*
2. <30 years
3. 30-39 years
4. 40-49 years
5. 50-59 years
6. ≥60 years
7. Are you: *(Single-choice question)*
8. Male
9. Female
10. What’s your technical title? *(Single-choice question)*
11. Junior
12. Intermediate
13. Associate-senior
14. Senior
15. What’s your education level? *(Single-choice question)*
16. Specialty
17. Bachelor
18. Master
19. Doctor
20. How many years have you been employed? *(Single-choice question)*
21. <5 years
22. 5-9 years
23. 10-14 years
24. 15-19 years
25. >20 years
26. What’s the grade or level of your hospitals? *(Single-choice question)*
27. Grade III general hospital
28. Grade III specialist hospital for women and children
29. Grade II general hospital
30. Grade II specialist hospital for women and children
31. Private general hospital
32. Private specialist hospital for women and children
33. Primary healthcare center (Grade I)
34. Other healthcare center
35. What’s the department or ward you currently work in? *(Single-choice question)*
36. Neonatology
37. Neonatal intensive care unit
38. Pediatrics
39. Pediatric intensive care unit
40. Children's healthcare unit
41. Other pediatric departments

| **Part 2 Clinical experience in neonatal sepsis management** |
| --- |

1. How many months do you work in neonatal department (ward) per year? *(Single-choice question)*
2. <1
3. 1-3
4. 4-6
5. 7-9
6. 10-12
7. Seldom
8. How do you access the experience of neonatal sepsis antibiotics management? *(Multiple-choice question)*
9. Departmental team experience (e.g. internal SOP, clinical pathways or discussion)
10. Lectures or classes from neonatal or pediatric specialists
11. Published guidelines, consensus or medical textbook
12. Consultation or teaching by clinical pharmacists
13. Consultation or teaching by anti-infection specialists
14. Not accessible
15. Does your hospital have a multidisciplinary (MDT) team for the management of anti-infective treatment of neonatal sepsis, including anti-infective specialists, clinical pharmacists, microbiologists, etc.? *(Single-choice question)*
16. There is a well-established MDT team for neonatal sepsis in the hospital
17. MDT is performed through inviting formal medical consultations
18. Invite a familiar anti-infection specialist, clinical pharmacy or microbiologist through private communication
19. No MDT teams
20. Unclear
21. What’s the frequency of MDT discussion in sepsis neonatal antibiotic use management? *(Single-choice question)*
22. Daily
23. 4-5 times weekly
24. 2-3 times weekly
25. Weekly
26. Depends on the clinical requirement
27. No MDT discussion
28. Unclear

| **Part 3 Current implementation of extended infusion or continuous infusion of antibiotics in neonatal sepsis** |
| --- |

In this part, extended infusion (EI) is defined as 2-4 hours compared with intermittent infusion (0.5h), and continuous infusion (CI) is defined as more than 8 hours. You are invited to answer the following questions based on actual clinical practice experience.

1. Do you have experience in EI/CI of β-lactams and vancomycin in neonatal sepsis? *(Single-choice question)*
2. Yes
3. No
4. What are reasons of EI/CI of β-lactams and vancomycin in neonatal sepsis? *(Multiple-choice question)*
5. The patient has multiple risk factors and a high probability of serious infections, such as multi-drug resistance infections
6. Blood culture results suggest that the bacteria are intermediate or resistance to β-lactams or vancomycin
7. Previous multiple antibiotics are ineffective, the efficacy may be improved by EI/CI administration
8. Routine EI/CI of β-lactams and vancomycin experience
9. Unclear
10. How do you obtain the source of EI/CI administration in neonatal sepsis? *(Multiple-choice question)*
11. Clinical practice guidelines or expert consensus
12. Lectures from anti-infection specialists
13. Lectures from clinical pharmacists
14. Academic conferences
15. Clinical pathways or guidance within the department
16. Medical textbook or drug instructions
17. Experience from consulting other pediatrians
18. Unclear
19. How long have you prolonged the infusion time of β-lactams in the management of neonatal sepsis? *(Multiple-choice question)*
20. Extended to 1 hour
21. Extended to 2 hours
22. Extended to 3 hours
23. Extended to 4 hours
24. Continuous to more than 8 hours
25. Unclear
26. How long have you prolonged the infusion time of vancomycin in the management of neonatal sepsis? *(Multiple-choice question)*
27. Extended to 2 hours
28. Extended to 3 hours
29. Extended to 4 hours
30. Continuous to more than 8 hours
31. Unclear
32. Which of the following drugs have you used an extended infusion (2-4h) regimen for? *(Multiple-choice question)*
33. Benzylpenicillin
34. Amoxicillin
35. Ampicillin
36. Oxacillin
37. Cloxacillin
38. Amoxicillin-clavulanate
39. Ampicillin-sulbactam
40. Piperacillin-tazobactam
41. Cefazolin
42. Cefuroxime
43. Ceftriaxone
44. Cefotaxime
45. Ceftizoxime
46. Ceftazidime
47. Ceftazidime-avibactam
48. Cefoperazone-sulbactam
49. Cefepime
50. Ceftaroline
51. Latamoxef
52. Meropenem
53. Imipenem-cistatin
54. Biapenem
55. Ertapenem
56. Vancomycin
57. No experience
58. Unclear
59. Which of the following drugs have you used a continuous infusion (>8h) regimen for? *(Multiple-choice question)*
60. Benzylpenicillin
61. Amoxicillin
62. Ampicillin
63. Oxacillin
64. Cloxacillin
65. Amoxicillin-clavulanate
66. Ampicillin-sulbactam
67. Piperacillin-tazobactam
68. Cefazolin
69. Cefuroxime
70. Ceftriaxone
71. Cefotaxime
72. Ceftizoxime
73. Ceftazidime
74. Ceftazidime-avibactam
75. Cefoperazone-sulbactam
76. Cefepime
77. Ceftaroline
78. Latamoxef
79. Meropenem
80. Imipenem-cistatin
81. Biapenem
82. Ertapenem
83. Vancomycin
84. No experience
85. Unclear
86. Please choose the stability of the following β-lactams and vancomycin at room temperature (25 ℃). *(Single-choice question)*

*(Each drug has 5 choices: <2h; 2-4h; 4-12h; >12h; Unclear)*

1. Amoxicillin
2. Cloxacillin
3. Ampicillin-sulbactam
4. Piperacillin-tazobactam
5. Cefotaxinme
6. Ceftazidime
7. Cefepime
8. Meropenem
9. Imipenem-cistatin
10. Vancomycin
11. Do you administrate loading dose of β-lactams or vancomycin to achieve rapid plasma therapeutic concentrations in severe infected children? *(Single-choice question)*
12. Routine loading dose
13. Selective administration of loading dose according to the patient's condition and severity of infection
14. No loading dose experience
15. Unclear
16. What’s your attitude towards EI/CI administration according to clinical observation? *(Single-choice question)*
17. No definitive conclusions on its efficacy because of limited experience
18. It had achieved better clinical improvement
19. There was inconsistency in the efficacy based on various cases
20. No better clinical improvement was observed using EI/CI administration
21. Unclear

| **Supplementary material 2 Multivariate logistic regression of factors associated with the implementation of EI/CI of β-lactams and vancomycin** | | | | | |
| --- | --- | --- | --- | --- | --- |
| Factors | EI/CI implementation (N=473) | No EI/CI implementation (N=1028) | Multivariate logistic regression | | |
|  |  |  | OR | 95%CI | P |
| **Gender** | | | | | |
| Women (Ref) | 349 (73.78%) | 787 (76.56%) |  |  |  |
| Men | 124 (26.22%) | 241 (23.44%) | 1.166 | 0.879-1.547 | 0.287 |
| **Age** | | | | | |
| <30 (Ref) | 36 (7.61%) | 111 (10.80%) |  |  |  |
| 30-39 | 176 (37.21%) | 357 (34.73%) | 1.599 | 0.818-3.124 | 0.170 |
| 40-49 | 164 (34.67%) | 346 (33.66%) | 1.448 | 0.614-3.142 | 0.397 |
| 50-59 | 90 (19.03%) | 202 (19.65%) | 1.234 | 0.475-3.209 | 0.666 |
| ≥60 | 7 (1.48%) | 12 (1.17%) | 0.920 | 0.234-3.616 | 0.905 |
| **Technical title** | | | | | |
| Junior (Ref) | 85 (17.97%) | 222 (21.60%) |  |  |  |
| Intermediate | 140 (29.60%) | 341 (33.17%) | 0.824 | 0.517-1.313 | 0.415 |
| Associate-senior | 127 (26.85%) | 273 (26.56%) | 0.850 | 0.463-1.560 | 0.599 |
| Senior | 121 (25.58%) | 192 (18.68%) | 1.164 | 0.566-2.395 | 0.679 |
| **Education** | | | | | |
| Specialties | 15 (3.17%) | 71 (6.91%) | 1.056 | 0.546-2.044 | 0.871 |
| Bachelor (Ref) | 261 (55.18%) | 755 (73.44%) |  |  |  |
| Master | 143 (30.23%) | 159 (15.47%) | 1.581 | 1.151-2.172 | **0.005** |
| Doctor | 54 (11.42%) | 43 (4.18%) | 1.903 | 1.161-3.121 | **0.011** |
| **Years of employment (years)** | | | | | |
| <5 (Ref) | 40 (8.46%) | 92 (8.95%) |  |  |  |
| 5-9 | 79 (16.70%) | 159 (15.47%) | 1.077 | 0.555-2.092 | 0.827 |
| 10-14 | 89 (18.82%) | 210 (20.43%) | 1.005 | 0.474-2.129 | 0.990 |
| 15-19 | 82 (17.34%) | 141 (13.72%) | 1.392 | 0.565-3.428 | 0.472 |
| >20 | 183 (38.69%) | 426 (41.44%) | 0.895 | 0.340-2.360 | 0.823 |
| **Hospital grade** | | | | | |
| Grade I | 19 (4.02%) | 121 (11.77%) | 0.614 | 0.160-2.352 | 0.477 |
| Grade II | 69 (14.59%) | 319 (31.03%) | 0.610 | 0.433-0.858 | **0.004** |
| Grade III (Ref) | 385 (81.40%) | 588 (57.20%) |  |  |  |
| **Hospital type** | | | | | |
| Primary health care center (Ref) | 16 (3.38%) | 108 (10.51%) |  |  |  |
| General hospital | 275 (58.14%) | 671 (65.27%) | 0.739 | 0.173-3.182 | 0.685 |
| Specialist hospital for women and children | 182 (38.48%) | 249 (24.22%) | 0.991 | 0.228-4.309 | 0.991 |
| **Department (ward)** | | | | | |
| Neonatology | 213 (45.03%) | 411 (39.95%) | 1.707 | 0.903-3.227 | 0.100 |
| Neonatal intensive care unit | 133 (28.12%) | 112 (10.89%) | 2.864 | 1.351-5.654 | **0.002** |
| Pediatrics | 87 (18.39%) | 288 (28.02%) | 1.452 | 0.802-2.628 | 0.219 |
| Pediatric intensive care unit | 4 (0.85%) | 4 (0.39%) | 3.243 | 0.627-16.776 | 0.160 |
| Children's healthcare unit | 13 (2.75%) | 56 (5.45%) | 1.542 | 0.688-3.459 | 0.292 |
| Other pediatric departments (Ref) | 23 (4.86%) | 157 (15.27%) |  |  |  |
| **Months in neonatal department (ward) per year** | | | | | |
| <1 | 17 (3.59%) | 90 (8.75%) | 0.826 | 0.419-1.627 | 0.58 |
| 1-3 | 29 (6.13%) | 75 (7.30%) | 1.340 | 0.696-2.579 | 0.382 |
| 4-6 | 58 (12.26%) | 58 (5.64%) | 2.622 | 1.398-4.918 | **0.003** |
| 7-9 | 31 (6.55%) | 48 (4.67%) | 1.451 | 0.713-2.953 | 0.305 |
| 10-12 | 299 (63.21%) | 520 (50.58%) | 1.390 | 0.791-2.442 | 0.253 |
| Seldom (Ref) | 39 (8.25) | 237 (23.05%) |  |  |  |
| **The way accessing the experience of neonatal sepsis antibiotics management** | | | | | |
| Departmental team experience |  |  |  |  |  |
| Yes | 358 (75.69%) | 614 (59.73%) | 1.041 | 0.761-1.423 | 0.802 |
| No (Ref) | 115 (24.31%) | 414 (40.27%) |  |  |  |
| Lectures or classes from neonatal or pediatric specialists |  |  |  |  |  |
| Yes | 19 (4.02%) | 78 (7.59%) | 1.447 | 0.987-2.121 | 0.058 |
| No (Ref) | 454 (95.98%) | 950 (92.41%) |  |  |  |
| Published guidelines, consensus or medical textbook |  |  |  |  |  |
| Yes | 67 (14.16%) | 140 (13.62%) | 0.924 | 0.672-1.270 | 0.627 |
| No (Ref) | 406 (85.84%) | 888 (86.38%) |  |  |  |
| Consultation or teaching by clinical pharmacists |  |  |  |  |  |
| Yes | 2 (0.42%) | 7 (0.68%) | 1.139 | 0.858-1.511 | 0.369 |
| No (Ref) | 471 (99.58%) | 1021 (99.32%) |  |  |  |
| Consultation or teaching by anti-infection specialists |  |  |  |  |  |
| Yes | 0 (0.00%) | 5 (0.49%) | 1.059 | 0.797-1.408 | 0.693 |
| No (Ref) | 473 (100.00%) | 1023 (99.51%) |  |  |  |
| Not accessible |  |  |  |  |  |
| Yes | 27 (5.71%) | 184 (17.90%) | 0.713 | 0.433-1.173 | 0.183 |
| No (Ref) | 446 (94.29%) | 844 (82.10%) |  |  |  |
| **MDT team in neonatal antibiotic use** | | | | | |
| There is a well-established MDT team for neonatal sepsis in the hospital | 76 (16.07%) | 68 (6.61%) | 1.886 | 1.090-3.265 | **0.023** |
| MDT is performed through inviting formal medical consultations | 178 (37.63%) | 239 (23.25%) | 1.386 | 0.881-2.182 | 0.158 |
| Invite a familiar anti-infection specialist, clinical pharmacy or microbiologist through private communication | 80 (16.91%) | 169 (16.44%) | 1.088 | 0.694-1.707 | 0.714 |
| Unclear | 113 (23.89%) | 437 (42.51%) | 1.165 | 0.610-2.228 | 0.643 |
| No MDT team (Ref) | 26 (5.50%) | 115 (11.19%) |  |  |  |
| **The frequency of MDT discussion in antibiotic use management** | | | | | |
| Daily | 15 (3.17%) | 15 (1.46%) | 2.462 | 1.011-5.992 | **0.047** |
| 4-5 times weekly | 13 (2.75%) | 11 (1.07%) | 2.725 | 1.028-7.226 | **0.044** |
| 2-3 times weekly | 24 (5.07%) | 24 (2.33%) | 1.973 | 0.938-4.152 | 0.073 |
| Weekly | 47 (9.94%) | 49 (4.77%) | 1.941 | 1.059-3.558 | **0.032** |
| Depends on the clinical requirement | 240 (50.74%) | 386 (37.55%) | 1.272 | 0.828-1.954 | 0.273 |
| Unclear | 99 (20.93%) | 378 (36.77%) | 1.307 | 0.715-2.387 | 0.384 |
| No MDT discussion (Ref) | 35 (7.40%) | 165 (16.05%) |  |  |  |
